# Assessing the Potential Distributions of the Invasive Mosquito Vector *Aedes albopictus* and Its Natural *Wolbachia* Infections in México

**DOI:** 10.3390/insects12020143

**Published:** 2021-02-07

**Authors:** David A. Moo-Llanes, Teresa López-Ordóñez, Jorge A. Torres-Monzón, Clemente Mosso-González, Mauricio Casas-Martínez, Abdallah M. Samy

**Affiliations:** 1 Centro Regional de Investigación en Salud Pública (CRISP), Instituto Nacional de Salud Pública (INSP), Tapachula, Chiapas 30700, Mexico; davidmooll@gmail.com (D.A.M.-L.); tlordonez@insp.mx (T.L.-O.); jatorres@insp.mx (J.A.T.-M.); clemente.mosso@insp.mx (C.M.-G.); 2 Entomology Department, Faculty of Science, Ain Shams University, Abbassia, Cairo 11566, Egypt

**Keywords:** *Aedes albopictus*, *Wolbachia* spp., distribution, *kuenm*, ecological niche modeling, México

## Abstract

**Simple Summary:**

This study updated the potential distribution of the Asian tiger mosquito *Aedes albopictus* in México and provided estimates to model uncertainty. We also assessed the potential distribution of natural *Wolbachia* infections in *Ae. albopictus* in México to map areas with circulation potential of *Wolbachia*. The distribution of *Ae. albopictus* covered the states across Northern México, the Gulf of México, the Pacific Coast of México, Central México, and the southeast of México. The ecological niche model of the *Wolbachia* infections anticipated its occurrence in the southeast of México, the Chiapas border with Guatemala, and Veracruz. While these results can prioritize vector surveillance and control programs for decision-makers, it is still necessary to establish active surveillance programs to validate the ecological niche of natural *Wolbachia* infections in *Ae. albopictus* populations in México.

**Abstract:**

The Asian tiger mosquito *Aedes albopictus* is currently the most invasive vector species, with a widespread global distribution. *Aedes albopictus* is the potential vector of diverse arboviruses, including Zika and dengue. This study updated the ecological niche model of *Ae. albopictus* and inferred the potential distribution of natural *Wolbachia* infections in *Ae. albopictus* in México. The ecological niche models were constructed based on diverse model settings to better estimate the potential distributions and uncertainty indices of both *Ae. albopictus* and its natural *Wolbachia* infections in México. The distribution of *Ae. albopictus* covered the states across Northern México, the Gulf of México, the Pacific Coast of México, Central México, and the southeast of México. The ecological niche model of the natural *Wolbachia* infections in *Ae. albopictus* populations anticipated the occurrence of natural *Wolbachia* infections in the southeast of México, the Chiapas border with Guatemala, and Veracruz. These results can be used to prioritize vector surveillance and control programs in México for strategic and future decision-making; however, it is still necessary to establish active surveillance programs to assess model predictions based on the independent sampling of *Ae. albopictus* from different invasion zones in México. Finally, vector surveillance should also screen the natural *Wolbachia* infections in *Ae. albopictus* to validate *Wolbachia* predictions across México, particularly in the southeast of México.

## 1. Introduction

The Asian tiger mosquito, *Aedes albopictus* (Skuse 1894) (Diptera: Culicidae), originated in Asia and is now distributed throughout tropical and subtropical regions [1]. This mosquito is an invasive species since it has successfully colonized many regions outsides its native habitats [2]. *Aedes albopictus* is also established at temperate latitudes in Europe and North America [1]. The mosquito *Ae. albopictus* invaded the Americas in 1985 via two main independent routes: United States and Brazil [3,4]. *Aedes albopictus* was first reported in Coahuila, México in 1993 [5]. Today, *Ae. albopictus* has been identified in 17 Mexican states, including Campeche, Chiapas, Coahuila, Hidalgo, México, Morelos, Nuevo León, Oaxaca, Puebla, Querétaro, Quintana Roo, San Luis Potosí, Sinaloa, Tabasco, Tamaulipas, and Veracruz y Yucatán [6,7,8,9,10,11,12,13,14]. The global spread of *Ae. albopictus* is of particular public health concern, particularly as the species is a potential vector of 22 arboviruses, including dengue, yellow fever, Chikungunya, and Zika [15]. México reported a total of 11,200 and 5667 cases of chikungunya and Zika in 2015, respectively [16,17].

Novel analytical tools were developed to calibrate and project the potential distribution of the species based on machine learning algorithms [18]. These tools estimate the distributional potential of the species by correlating the species occurrences and the environmental covariates. Ecological niche modeling has been used to project the geographic distributional potential of numerous vectors and vector-borne diseases (VBDs). These diseases included dengue, Zika, chikungunya [1,19], Chagas diseases [20,21], leishmaniasis [22], and malaria [10].

*Wolbachia* spp. are extremely common bacteria that occur naturally in 60% of insect species, including some mosquitoes, fruit flies, moths, dragonflies, and butterflies. *Wolbachia* spp. can invade insect populations using cytoplasmatic incompatibility and provide new strategies for controlling mosquito-borne diseases, such as dengue fever [23]. Cytoplasmic incompatibility causes a significant reduction in brood hatch and promotes the spread of the maternally inherited *Wolbachia* infection into the host population. *Wolbachia*-infected females live longer, produce more eggs, and have higher hatching rates in compatible crosses [24]. The World Mosquito Program released males and females of the *Wolbachia*-infected mosquito *Ae. aegypti* to protect global communities from mosquito-borne diseases [25]. *Wolbachia*-infected mosquitoes breed with the wild mosquito population until a higher percentage of mosquitoes carrying *Wolbachia* has been reached. These infected mosquitoes have a reduced ability to transmit pathogens such as Zika, dengue, Chikungunya, and yellow fever viruses to human populations [25]. *Wolbachia*-carrying mosquitoes were released across La Paz city, Baja California Sur, México in January 2019 to offer a long-term sustainable alternative to the currently available disease-control strategies [25]. There are only three reports of *Ae. albopictus* infected with the bacterium *Wolbachia* in México [26,27,28]. Another additional report identified the coinfection of the native *Ae. albopictus* populations with both *wAlbA* and *wAlbB* strains of *Wolbachia* [27].

*Aedes albopictus* is continuously invading new areas in México. Therefore, we need to continuously update the ecological niche model (ENM) of this important vector by considering the availability of new data and development of the ecological niche modeling “toolkit” to better calibrate and evaluate these models. As part of gaining a better understanding of the distributional potential of *Ae. albopictus* in México, this study had three objectives: (1) to update the ENM of *Ae. albopictus* and estimate the population at risk; (2) to validate the ENM from 2015 to 2020 to assess the temporal differences in the ecological niches; and finally, (3) to construct the ENM of natural *Wolbachia* infections based on occurrences of *Wolbachia*-infected *Ae. albopictus* in México. The results of this study could be used to prioritize vector control measures in México and delineate the target populations of *Ae. albopictus* infected with *Wolbachia* spp. for future decision-making.

## 2. Materials and Methods

### 2.1. Ecological Niche Modeling of Ae. albopictus in México

#### 2.1.1. Database

**Figure 1:**
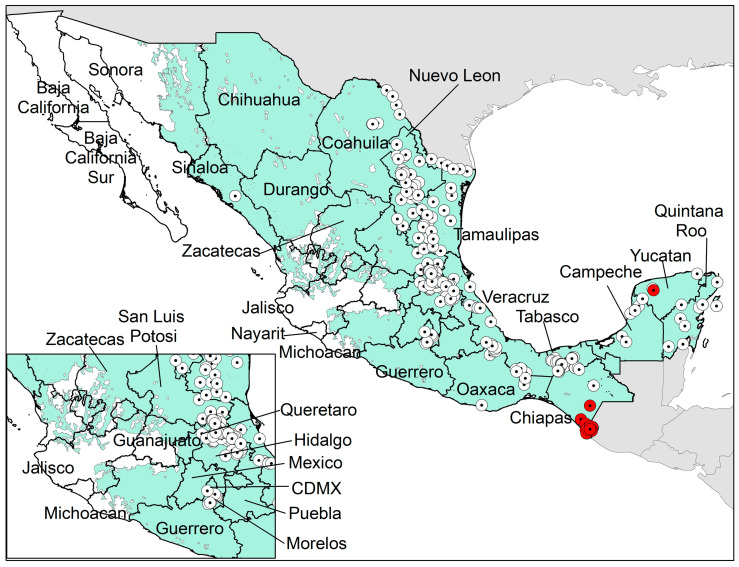
**A map of México showing the boundaries of the Mexican states.** The white dotted circles represent the occurrence records of *Aedes albopictus* available for the model calibration. The red dotted circles represent the occurrence records of natural *Wolbachia* infections in *Ae. albopictus* populations. The green background depicts the accessible areas where the *Ae. albopictus* model was calibrated. The square in the bottom left corner of the map presents a close-up of the central states of México.

A Mexican database of *Ae. albopictus* occurrences were obtained from previous literature [6,7,8,9,11,12,13,14,29]. These occurrences presented sites of adult sampling obtained from Pech-May et al. [9], and records from all recent surveillance programs of *Ae. albopictus* in México from 2015 to 2020 (Figure 1). A total of 316 occurrences were included in the original dataset. We eliminated duplicated occurrences and reduced the effects of spatial autocorrelation by thinning occurrence records via a distance filter of 5 km between records using the *spThin* R package [30]. The final database included 231 occurrence records. We randomly split occurrence records into three subsets using the “random k-fold” method: 65% of occurrences for model calibration, 25% of occurrences for internal testing, and the remaining 10% of occurrences for final evaluation [22]. The latter method partitions occurrence localities randomly into a user-specified number of (k) bins as described in detail in the previous protocol [31].

#### 2.1.2. Accessible Area (M)

The accessible area “***M***” is an important component in the biotic, abiotic, and movement (***BAM***) diagram, defining the main parameters in constructing the species ecological niche model [32]. A 200 km radius buffer was created around each occurrence point to extend the limits of the entire calibration region considering the broad invasion potential of *Ae. albopictus*. The selection of this radius buffer was based on the spatial resolution of environmental variables and the environmental heterogeneity present in areas where the species occur. Accessible area “***M***” represents the areas to which a species has had access over a relevant time-period because of its movement and colonizing capacities and the structure of barriers and distances [32]. Each occurrence record was subsequently overlaid on the ecoregion shapefile to assess the concordance between the species occurrence and a particular ecoregion [33], as described in a previous study [20]. We defined the ecoregions as relatively large units of land containing a distinct assemblage of natural communities and species with boundaries that approximated the original extent of natural communities before major land-use changes [33].

#### 2.1.3. Bioclimatic Variables

Sixteen variables were used to construct the ENM of *Ae. albopictus* in México. These variables included fifteen bioclimatic variables from the WorldClim version 2 and the elevation. We used WorldClim 2 because WorldClim data performed substantially better than other available climatic data in different modeling purposes (http://www.worldclim.org; ref. [34]). We excluded four variables from the bioclimatic variables (Bio 8, Bio 9, Bio 18, and Bio 19) owing to their known spatial artifacts, following the protocol implemented in previous similar studies [35,36]. The elevation variable was also selected in light of its important contribution in constructing the species ecological niche model [20]. This variable was downloaded from the Consultative Group on International Agricultural Research-Consortium for Spatial Information (CGIAR-CSI) available at http://cgiar-csi.org/data/srtm/. All these variables had a spatial resolution of 30 arc-seconds (0.008333° ≈ 1 km). We used the iterative jackknife function implemented in the software MaxEnt 3.4.1 [37] to identify four candidate sets of predictors to improve the model calibration process of *Ae. albopictus* by reducing the spatial autocorrelation of presence data and the multi-collinearity of the bioclimatic variables [22]. Variables were selected considering their contribution to models and their collinearity. Final sets of variables included 16 variables in set 1, 15 in set 2, 8 in set 3, and 10 in set 4 (Table 1): (a) set 1 (15 bioclimatic variables from WorldClim and elevation); (b) set 2 (15 bioclimatic variables from WorldClim); (c) set 3 (7 bioclimatic variables and elevation); and (d) set 4 (9 bioclimatic variables and elevation).

#### 2.1.4. Ecological Niche Modeling of *Ae. albopictus*

We constructed ENM using the maximum entropy algorithm implemented in MaxEnt version 3.4.1 via the *kuenm* R package [38]. We created candidate models by combining four sets of environmental variables, 17 values of regularization multipliers (0.1–1 with intervals of 0.1, 2–6 with intervals of 1, and 8 and 10), and all 29 possible combinations of 5 feature classes (linear = l, quadratic = q, product = p, threshold = t, and hinge = h) [38]. The best candidate model was selected based on three criteria: 1) significance, 2) performance, and 3) the Akaike information criteria (AIC): AICc, delta AICc, and AICc weights. Statistical significance was determined by a bootstrap resampling of 50% of testing data, and probabilities were assessed by direct count of the proportion of bootstrap replicates for which the AUC ratio was ≤1.0. Performance was measured using omission rates, which indicate how well the models constructed with training data can anticipate test occurrences based on a maximum allowable omission error rate of 5%, assuming that up to 5% of occurrence data may include errors that misrepresented environmental values. We followed the criteria from a previous study [38] for selecting the final model, evaluating the model, and assessing extrapolation risk. We created the final models of *Ae. albopictus* using 10 replicates by bootstrap, with logistic outputs, and transferred these models from the accessible area “***M***” to the projection area “***G***”.

### 2.2. Extrapolation Risk and Uncertainty Map of Ae. albopictus

To identify extrapolation risk in the model transfers, we performed a mobility-oriented parity (MOP) analysis comparing the environmental breadth of predictors within “***M***” (10% reference points sampled) with that in the projection area using the MOP function [39] available in the *kuenm* R package [38]. The risk of extrapolation analysis defines the areas with strict extrapolation (i.e., places where environmental conditions are non-analogous to those in areas across which the models were calibrated) to avoid the risk of over-prediction in non-analogous environments. The uncertainty maps were constructed using the MOP analysis and the standard deviation (SD) obtained from the final models in *kuenm*. The MOP raster output was reclassified into five categories; the first category represented a strict extrapolation (i.e., zero value), and the fifth category represented the highest environmental similarities between calibration and projection areas. The standard deviation raster was reclassified into five categories too; category “1” represented the lowest values of SD, and category “5” represented the highest values of SD. We subsequently combined the two classified rasters as an estimate for the uncertainty index; the final raster output was reclassified into three categories: low, medium, and high, to present lower, medium, and higher values of uncertainty index, respectively.

### 2.3. The Total Human Population at Risk of Contact with Ae. albopictus

The total human population growth rate of México was generated using projections of fertility, mortality, and international migration [40]. The National Population Council proposed a 30% increase in the population growth rate from 2010 to 2020 in México [41]. The Mexican human population from the 2010 census was 112,336,537 inhabitants [42]. After obtaining the ENM 2020, human population projections were calculated and the human population at risk of contact with *Ae. albopictus* was estimated based on two categories: rural population (communities < 10,000 inhabitants) and urban population (communities > 10,000 inhabitants) [42].

### 2.4. Validation of the ENM of Ae. albopictus from 2015 to 2020

The ENM of *Ae. albopictus* for 2015 was constructed using the database of the previous study [9] with 198 unique occurrences. We used a single set of the same 13 variables used in the previous study [9] to replicate the previous constructed ENM [9]: annual mean temperature, temperature seasonality, temperature annual range, annual precipitation, precipitation of wettest month, precipitation of driest month, precipitation seasonality, aspect, slope, topographic index, and elevation [9]. The steps of estimating the accessible area “***M***” and constructing the ENM followed the previously described methodology using the *kuenm* package [38]. This model was denoted as “Pech-May et al. [9] ENM”; while the ENM obtained in this study was denoted as “2020 ENM”. We selected a threshold that predicted the presence of 90% of occurrences. We then converted the values that were greater or equal to this threshold value into “1” (presence) and the values less than this threshold value into “0” (absence) to get a binary map of the distribution [42]. Model validation is often an integral part of ENM development; this consists of evaluating the model based on independent records that were not included in the previous model calibration step. The latter evaluation approach identified the proportion of correctly predicted presence records and thus the quantification of omission errors [43]. We also evaluated the geographical projection of the three variants of ecological niches: (a) conserved niche was defined as the ecological niche that remains constant in both models: Pech-May et al. ENM [9] and 2020 ENM, (b) gain of niche of Pech-May et al. [9] ENM compared to that of 2020 ENM, and (c) loss of niche of Pech-May et al. [9] ENM compared to that of 2020 ENM. The ENMs in both models were calculated in pixels occupied/all pixels.

We further visualized the environmental space of both models in the software Niche Analyst (NicheA) version 3.0 [44] available at http://nichea.sourceforge.net/. This software allows visualization of environmental distribution as a minimum volume ellipsoid in three dimensions of environmental space (i.e., the first three principal components out of the principal component analysis of the 13 environmental variables described above). We calculated the niche overlap using the kernel density function implemented in the *ecospat* package in R [45] to estimate the density of the species in environmental space as per a previous protocol [46].

### 2.5. ENM of Natural Wolbachia Infections in Ae. albopictus Populations

The occurrence records of natural *Wolbachia* infections in the host *Ae. albopictus* were obtained from previously published studies in México [26,27]. The natural *Wolbachia* infections in mosquito populations were determined based on PCR amplification of a 600 bp fragment corresponding to the *wsp* gene [27]. The previous studies [26,27] identified natural *Wolbachia* infections in 21 unique sampling sites across México. These unique occurrences were randomly divided into three categories: model calibration (65%), internal testing (25%), and model evaluation (10%) using the methodology protocol implemented in our previous study [22]. For creating the accessible area “***M***” [32], we used a binary map delineating the species distribution described above. We considered the accessible area “***M***” as the space in the hyper-volume, where *Ae. albopictus* had a prediction of 90% in México. We used the four sets of climatic variables described above to construct the ecological niche model of natural *Wolbachia* infections (Table 1). We used the MaxEnt algorithm via the *kuenm* R package [38] to construct ENM as previously described above in the previous sections. To identify extrapolation risk in the model transfers, we performed MOP analysis as previously described using the *kuenm* R package. The uncertainty map was constructed using MOP analysis and the standard deviation (SD) raster obtained from the final models in *kuenm*. These rasters were classified as previously described above in *Ae. albopictus* model. The two classified outputs were combined to provide a proxy of the uncertainty index. This final raster was reclassified into three categories: low, medium, and high, to represent lower, medium, and higher values of uncertainty index, respectively.

## 3. Results

### 3.1. Distributional Potential of Ae. albopictus by 2020 in México

**Figure 2:**
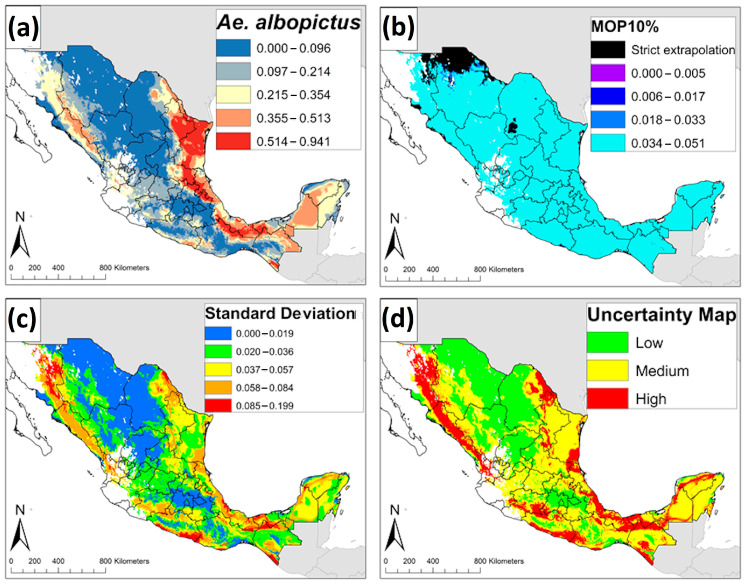
**Ecological niche model and uncertainty maps of *Aedes albopictus* in México**. (**a**) Ecological niche model of *Aedes albopictus* in México. (**b**) Extrapolation risk in projecting the model of *Aedes albopictus* from the calibration area to a projection area based on a mobility-oriented parity (MOP) 10%. (**c**) Standard deviation map; (**d**) Uncertainty map of *Aedes albopictus* prediction.

We used four sets of variables in estimating the ENM of *Ae. albopictus* (Table 1). A total of 1972 candidate models were built for *Ae. albopictus*, however, 1479 of these models were statistically significant. Finally, only one model met the three selection criteria and was identified as the best candidate model based on its performance (Table 1). Precipitation was the primary contributor to the *Ae. albopictus* model; precipitation of the driest quarter (Bio 17), precipitation of the driest month (Bio 14), and annual precipitation (Bio 12) were the most influential variables in calibrating the *Ae. albopictus* model. The ENM of *Ae. albopictus* in México anticipated its distribution on the Gulf Coast of México, including the Yucatán Peninsula. The distribution of *Ae. albopictus* also covered the states of the Pacific Coast (Sonora, Sinaloa, Nayarit, Jalisco, Michoacán, Guerrero, Oaxaca, and Chiapas) and Central México (Puebla, Morelos, México, and Guanajuato) (Figure 2a). The MOP results suggested high levels of environmental similarities in all areas in México, except parts of Northern México (e.g., Chihuahua, Sonora, and Coahuila) where strict extrapolation occurred (Figure 2b). The highest values of standard deviation (Figure 2c) and uncertainty index (Figure 2d) corresponded to the southeast of México (Yucatán, Campeche, Chiapas, Veracruz, and Oaxaca), the Pacific Coast (Guerrero), and Northern México (Coahuila, Sonora, and Sinaloa).

About 13,690,890 inhabitants in rural populations and 18,735,366 inhabitants in urban populations were estimated to be at risk of mosquito contact in 2010. The human population at risk of mosquito bites increased to 16,026,995 inhabitants in rural populations and 21,664,851 inhabitants in urban populations in 2020.

### 3.2. Validation of the Distributional Potential of Ae. albopictus in México

**Figure 3:**
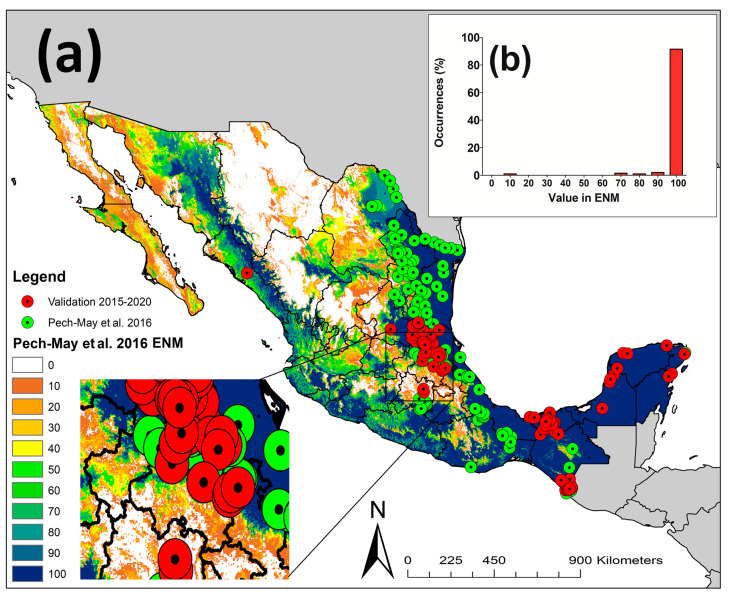
**Validation of the Pech-May et al. [9] ecological niche model (ENM) of *Aedes albopictus* in México.** (**a**) The calibration (dotted green circles) and validation (dotted red circles) occurrences of *Ae. albopictus* overlap with the Pech-May et al. [9] ENM; the values (0–100) represent the probability of *Ae. albopictus* occurrence in a particular area multiplied by 100. (**b**) The relationship between the percentage of validation occurrences on the vertical axis and the model prediction values on the horizontal axis. The red bars present the percentage of validation occurrence records for each prediction value (i.e., this shows that most validation records occurred in the upper 10th percentile of the predicted suitability values). The square at the bottom left corner of the map presents a close-up map showing the overlap between the occurrence records and the prediction in the background.

**Figure 4:**
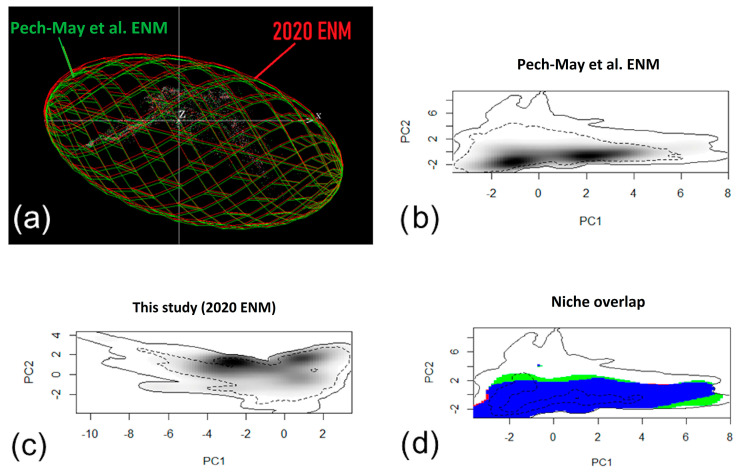
**Niche overlap between the Pech-May et al. [9] ecological niche model (ENM) and the 2020 ENM of *Aedes albopictus* in México.** (**a**) The ecological niche model is based on a minimum-volume ellipsoid in NicheA. (**b**) Occupied niche based on the Pech-May et al. [9] ENM. (**c**) Occupied niche based on the 2020 ENM. (**d**) Niche overlap of both ecological niches: blue depicts the niche overlap, red depicts the Pech-May et al. [9] ENM, and the green depicts the 2020 ENM.

**Figure 5:**
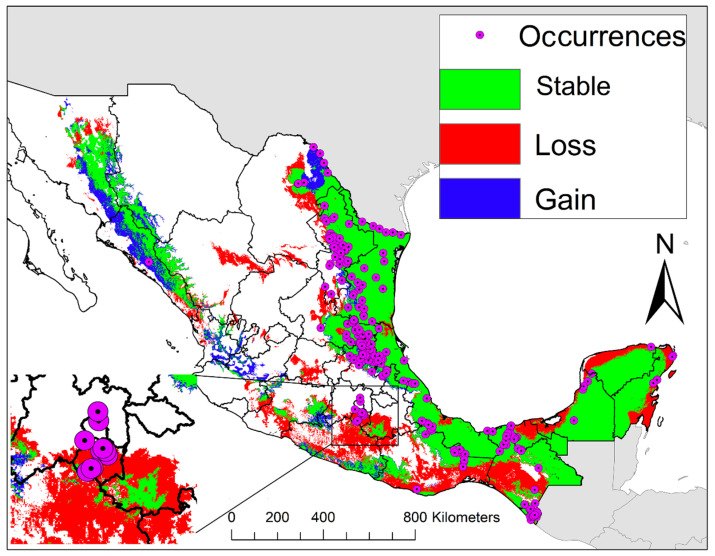
**Comparisons of ecological niche models of *Aedes albopictus* in México.** The relationships between the Pech-May et al. [9] ecological niche model and the 2020 ecological niche model. The green areas represent the areas where congruent predictions occurred between the two models, the red color represents the areas where *Aedes albopictus* is anticipated to occur by the Pech-May et al. [9] ENM only, and blue color represents the areas where *Ae. albopictus* is anticipated by the 2020 ENM only. The square at the bottom left corner of the map presents a close-up map showing the relationship between some of 2015–2020 occurrences and the Pech-May et al. [9] prediction.

The sensitivity of the Pech-May et al. [9] ENM was 191/198 = 0.96 compared to the sensitivity of the post-analysis validation (2015–2020) of 113/118 = 0.96 (Figure 3a). Most of the validation occurrences (91.41%) corresponded to a 100% predicted suitability of the Pech-May et al. [9] ENM (Figure 3b), compared to 94.06% of validation occurrences overlapped with areas of 100% predicted suitability in the 2020 ENM (S1 File). All validation occurrences occurred in the upper predicted suitability values of 2020 ENM (S1 File); however, some validation occurrences (1.21%) occurred in the lower 10% predicted suitability of the Pech-May et al. [9] ENM (Figure 3b). The niche overlap between both models was broad using both NicheA (Figure 4a) and kernel density (Figure 4d). The 2020 ENM also showed a broader niche than Pech-May et al. [9] ENM (Figure 4d). The overlap between the Pech-May et al. [9] ENM and 2020 ENM was 61.35% (646,560 km^2^), while the loss corresponded to 260,345 km^2^, and the gain corresponded to 98,481 km^2^ (Figure 5).

### 3.3. ENM of Natural Wolbachia Infections in the Host Ae. albopictus

**Figure 6:**
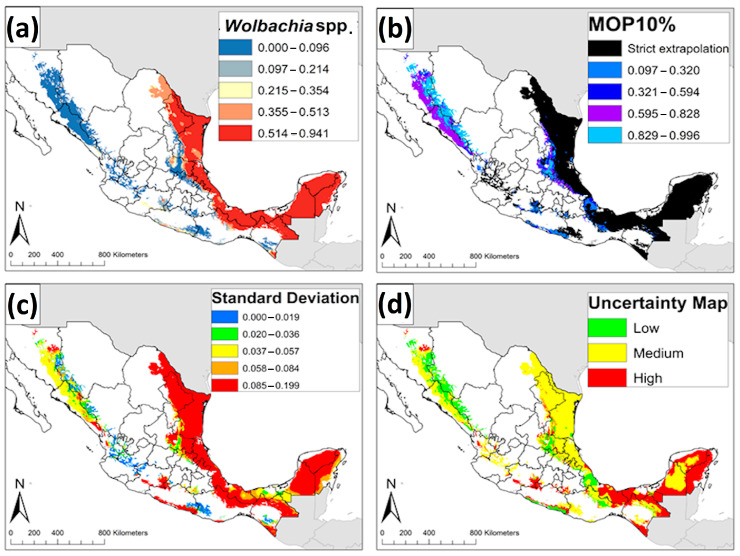
**Ecological niche model and uncertainty maps of natural *Wolbachia* infections in the host *Aedes albopictus* in México**. (**a**) Ecological niche model of natural *Wolbachia* infections in México. (**b**) Extrapolation risk in projecting the *Wolbachia* model from the calibration area to a projection area (mobility-oriented parity (MOP) 10%). (**c**) Standard deviation map; (**d**) Uncertainty map of *Wolbachia* prediction model.

The same four sets of variables were used in constructing the ENM of natural *Wolbachia* infections in the host *Ae. albopictus* (Table 1). A total of 1446 models were statistically significant; however, only 17 candidate models were statistically significant models meeting omission rate and AICc criteria (Table 1). The ENM of natural *Wolbachia* infections anticipated occurrence of *Wolbachia*-infected *Ae. albopictus* in most of the Yucatán Peninsula, Tabasco, north of Chiapas, the Chiapas border with Guatemala, and Veracruz (Figure 6a). The MOP results suggested high levels of environmental similarities in all areas in México except parts of the southeast of México (Yucatan Peninsula, Chiapas, Tabasco, and Veracruz), and Northern México (Tamaulipas, Nuevo León, and Coahuila) where strict extrapolation occurred (Figure 6b). The highest values of standard deviation (Figure 6c) and uncertainty index (Figure 6d) corresponded to the southeast of México (Yucatán, Campeche, Quintana Roo, Chiapas, Tabasco, and Veracruz), and Central México (Guerrero).

## 4. Discussion

The dispersal of invasive mosquitoes constitutes a dynamic ecological process that is strongly influenced by human activity on both local and global levels. Modification of the landscape, environmental pollution, the introduction of non-native species, and climate change are the main factors that cause important alterations in the bionomics of vectors over time. The Asian tiger mosquito *Ae. albopictus* has been identified in 17 states of México [6,7,8,9,10,11,12,13,14,28,29]. This mosquito species is considered an important invasive species since it has successfully colonized many sites outside its native habitats in Asia [2]. There are several advantages that increase the ability of the mosquito *Ae. albopictus* to adapt to new invasive habitats across the world: (a) eggs are more resistant to desiccation, which allows them to survive in inhospitable environments, in addition to favoring their transport via diverse human activities; (b) active transportation and human activities, which allow voluntarily or involuntarily transport from one place to another; (c) the presence of the aquatic and terrestrial stages help to streamline their transport [2,47]. In anthropized landscapes with intense human activity, the mosquito species initiate a process of adaptation to the new environmental and habitat conditions that can be reflected in different degrees of synanthropy between arbovirus vectors such as *Ae. aegypti* and *Ae. albopictus*.

The introduction of *Ae. albopictus* in the southern United States caused the rapid removal of *Ae. aegypti* in many places and the reduction of its distribution across the southeast of the country from Texas to Florida [48]. However, the vector competence of *Ae. albopictus* and *Ae. aegypti* in transmitting the dengue is different, public health programs should target the control of both vector species to reduce the transmission of dengue, even if the virus is potentially transmitted by *Ae. aegypti*. *Aedes albopictus* is a permanent resident of our region since its invasion of the Americas in the 1980s, indicating its increased associations in disease transmission across the region. The interactions of *Ae. albopictus* with *Ae. aegypti* and other mosquitoes can modify the vector capacity of the species involved in ways not always predictable [48].

The ecological niche models of Pech-May et al. [9] demonstrated a 79.7% coverage in México. The important overlap with the Asian niche model (i.e., ecological niche calibrated in Asia and projected to México) suggested a high potential for the species to disperse to sylvatic regions in México. Post-validation occurrences demonstrated the presence of *Ae. albopictus* in areas that previously reported a high probability based on a previous study [9], such as the Yucatan Peninsula. Currently, the potential invasion zones of *Ae. albopictus* in 2020 are unpredictable, and there are still too many behavioral, ecological, demographic, and genetic analyses concerning the competition that are being presented in each area. In addition to all these factors, it would still be necessary to assess the species occurrence and vector competence in rural and urban areas of México.

The new model of *Ae. albopictus* using all occurrences seems to have lower coverage than those proposed by the previous studies [9,49] and compared to those of the global models of *Ae. albopictus* [1,19,50]. Interestingly, this study benefited from the availability of more occurrence records in México to update the ecological niche of *Ae. albopictus* in México. Our study observed some limitations in the previous attempts of constructing the ecological niche of *Ae. albopictus* in México [9,49]. The previous modeling efforts of *Ae. albopictus* in México were associated with some sources of bias in constructing the ecological models, particularly if their available protocol had some limitations that were not suitable for the species, area, and extrapolation zones [51,52]. For example, a previous study [53] argued that the partial occurrence records are biased by an optimal ecological niche model, therefore, a complete sampling of all the possible occurrences of the species is indispensable. Here, we used a novel improved methodology and allowed different modeling settings [38] to construct the species ecological niche models based on calibration and evaluation of our models using a large number of updated variables that previously could not be used. We allowed the optimal parameterization based on the set of environmental variables, features, and multiple regularizations for better construction of the ecological niche model of the Asian tiger mosquito *Ae. albopictus*. It is worth mentioning that this methodology has also been applied in the field of vector-borne diseases, such as the *Lutzomyia longipalpis* complex and leishmaniasis vectors [22], and to infer the distribution of a *Rickettsia parkeri* pathogen transmitted by several tick species in America (personal communications Sokani Sanchez-Montes, UNAM). This study anticipated the potential distribution of *Ae. albopictus* in 2020 and identified areas of strict extrapolation where results should be interpreted with caution [39]. The climatic conditions were surprisingly analogous in calibration and projection areas; therefore, we can have a high degree of certainty in the potential distribution in México. Previous studies [9,49] did not provide any evidence for extrapolation risks and analogous climatic conditions between the different areas they evaluated. An early study [49] used the standard deviation as an approximation of the areas where care must be taken in the interpretations of the ecological niche model. Our maps presented an estimate of the uncertainty index based on both the MOP analysis (i.e., analogous versus non-analogous climatic conditions) and a map with the combined standard deviation values as a proxy of the uncertainty in the potential distribution areas. We agree with the previous studies [54,55] regarding the importance of assessing uncertainty indices with the potential distribution map for better interpretations of the prediction maps in the different invasion areas.

Interestingly, our study revealed the importance of precipitation variables as a limiting factor in calibrating the ecological niche model of *Ae. albopictus*. Several previous studies observed that precipitation was the most important predictor to assess the distributional potential of *Ae. albopictus* in diverse regions across the world, including México [1,19,56,57,58]. *Aedes albopictus* is a container-breeding mosquito, where drought and rainfall conditions can affect the aquatic ecosystem. For example, drought disrupts the aquatic ecosystem by increasing the larval density, which subsequently enhances interspecific competition and resource limitations. The latter drive increased larval development time and mortality, decreased adult longevity, and decreased adult size [58,59]. Another important finding from our study was the incongruence between the contribution of elevation as an important limiting factor in *Ae. albopictus* and natural *Wolbachia* infections models; *Ae. albopictus* was limited by covariates of precipitation, temperature, and elevation; however, the ecological niche of natural *Wolbachia* infections was not limited by elevation. This was an important finding, suggesting possibilities of natural *Wolbachia* infections in both lower and higher altitudes. The latter finding also raised the possibility of releasing *Wolbachia*-infected mosquitoes in plateau areas where arboviruses spread [60].

The World Mosquito Program uses *Wolbachia* to prevent the transmission of mosquito-borne viral diseases such as dengue, Zika, chikungunya, and yellow fever. There are several pieces of evidence from the international pilot studies that showed the importance of broad-release trials of *Wolbachia* to decrease the risk of arboviruses transmitted by *Ae. aegypti*. Multiple trials in various countries demonstrated that this control approach is a safe and an effective way to prevent the spread of diseases across entire cities and regions [25]. Two strategies using *Wolbachia* are available: (a) suppression (i.e., this strategy involves releasing a very large number of male mosquitoes carrying *Wolbachia*), and (b) replacement (i.e., this strategy requires releasing both male and female mosquitoes carrying *Wolbachia*). However, *Ae. aegypti* lacks these associations with *Wolbachia* in the field; *wAlbA* and *wAlbB* strains of *Wolbachia* were naturally identified from the vector *Ae. albopictus* [61,62]. The natural *Wolbachia* infections of *Ae. albopictus* was associated with a decrease of dengue virus transmission in mosquitoes from La Reunion Island [63]. In México, there are only three reports of *Wolbachia* infections in *Ae. albopictus* collected from different sites [26,27,28]. It is worth mentioning that these reports corresponded to the southeast region of México, specifically the Soconusco de Chiapas. The model of natural *Wolbachia* infections offered a proxy for the potential distribution of the naturally-infected populations of *Ae. albopictus* in México. The model anticipated higher probabilities of *Wolbachia* infections in *Ae. albopictus* in southeastern México, which corresponded to Yucatán, Campeche, Quintana Roo, Tabasco, and Chiapas. Perhaps this is a reason for the reduced populations of *Ae. albopictus* in the southeast of México. A recent study found *Ae. albopictus* naturally infected with *Wolbachia* in the Soconusco region, Chiapas [26]. These findings corroborate that the natural populations of *Ae. albopictus* in México and the World may be influenced by two aspects: (1) the nature of the interspecific competition and co-occurrence of *Ae. albopictus* and *Ae. aegypti*, and (2) the effect of *Wolbachia* infections on *Ae. albopictus* populations. This study addresses a new line of research. Predictions of the spatial distribution of natural *Wolbachia* infections in *Ae. albopictus* in México infer the geographic regions where the human populations could be protected from the medically important arboviruses transmitted by this species, particularly if the presence of *Wolbachia* reduces the arbovirus transmission and acts as a natural antiviral control agent. Our modeling efforts also provide some recommendations to determine the target priorities for *Wolbachia*-based control programs and release trials to save money and efforts for efficient control programs.

Finally, knowing the potential distribution of *Ae. albopictus* in México offers an overview of how to create vector control measures in each of the invasion zones. Likewise, this study stands out as one of the first to validate the ecological niche models of the key disease vector *Ae. albopictus* using newly updated sets of occurrences from recent surveillance programs in México. The new potential distribution of *Ae. albopictus* offers an opportunity to start with preventive measures in areas with a high probability of establishment in México. It is still necessary to validate all the areas in México with the probability of finding *Wolbachia* infection, mainly in the southeast of México. Interestingly, a recent study [28] identified natural coinfection of native populations of *Ae. albopictus* with two *Wolbachia* strains (*wAlbA* and *wAlbB*) in three suburban localities of the city of Merida, Yucatan. This latter study [28] estimated natural *Wolbachia* infections of 40% in local populations of *Ae. albopictus*. Therefore, our results can be applied in vector control measures in México for future decision-making. In summary, our future studies will consider further detailed mapping of *Wolbachia* strains, considering other additional host parameters and future climatic conditions.

## Figures and Tables

**Table 1:** Model performance under optimal parameters using sets of environmental predictors (SEP), statistically significant models (SSM), best candidate models (BCM), regularization multiplier (RM), features classes (FC), mean Area Under the Curve ratio (AUC.r), partial Receiver Operating Characteristic (p.ROC), omission rate 5% (O.rate 5%), Akaike information criterion corrected (AICc), delta Akaike information criterion corrected (∆AICc), Akaike information criterion corrected weight (AICc.W), number of parameters (#; summarizes the combination of environmental variables, multiple regularizations, and features other than 0 that provide information for the construction of the model based on lambdas), and candidate sets of environmental variables tested during calibration of the *Aedes albopictus* model in México. * q = quadratic; t = threshold; h = hinge; p = product.

SEP	SSM	BCM	RM	FC *	AUC.r	p.ROC	O.rate 5%	AICc	∆AICc	AICc.W	#
*Aedes albopictus*											
Set3	1479	1	3.0	qth	1.29	0.00	0.04	5941.66	0.00	0.79	35
Natural *Wolbachia* infections in *Ae. albopictus* populations											
Set2	1446	17	0.2	p	1.84	0.00	0.00	275.14	1.74	0.01	4
Candidate sets of environmental variables of *Ae. albopictus* and natural *Wolbachia* infections models											
**Set1**			**Set2**			**Set3**			**Set4**		
Bio 1, Bio 2, Bio 3, Bio 4, Bio 5, Bio 6, Bio 7, Bio 10, Bio 11, Bio 12, Bio 13, Bio 14, Bio 15, Bio 16, Bio 17, and elevation			Bio 1, Bio 2, Bio 3, Bio 4, Bio 5, Bio 6, Bio 7, Bio 10, Bio 11, Bio 12, Bio 13, Bio 14, Bio 15, Bio 16, and Bio 17			Bio 4, Bio 7, Bio 11, Bio 12, Bio 13, Bio 14, Bio 17, and elevation			Bio 1, Bio 4, Bio 5, Bio 6, Bio 7, Bio 12, Bio 13, Bio 14, Bio 15, and elevation		

Bio 1: Annual Mean Temperature; Bio 2: Mean Diurnal Range; Bio 3: Isothermality; Bio 4: Temperature Seasonality; Bio 5: Maximum Temperature of Warmest Month; Bio 6: Minimum Temperature of Coldest Month; Bio 7: Temperature Annual Range; Bio10: Mean Temperature of Warmest Quarter; Bio11: Mean Temperature of Coldest Quarter; Bio12: Annual Precipitation; Bio13: Precipitation of Wettest Month; Bio14: Precipitation of Driest Month; Bio15: Precipitation Seasonality; Bio16: Precipitation of Wettest Quarter; and Bio17: Precipitation of Driest Quarter.

## Data Availability

All data are publicly available in the manuscript text and its Ssupplementary materials. Occurrence records of *Ae. albopictus* and natural *Wolbachia* infections were deposited via the Figshare repository available in https://doi.org/10.6084/m9.figshare.13708318.

## Supplementary Materials

The following are available online at https://www.mdpi.com/2075-4450/12/2/143/s1, **S1 File**: Validation of the 2020 ecological niche model of *Aedes albopictus* in México.
