# Supplementary material for: Assessing the Potential Distributions of the Invasive Mosquito Vector Aedes albopictus and Its Natural Wolbachia Infections in México
Source: Insects. 2021 Feb 7;12(2):143. doi: 10.3390/insects12020143 (PMC7914640; doi:10.3390/insects12020143)

S1 File. Validation of the 2020 ecological niche model of *Aedes albopictus* in México. (**a**) The calibration (dotted green circles) and validation (dotted red circles) occurrences of *Ae. albopictus* overlap with the 2020 ENM; the values (0–100) represent the probability of *Ae. albopictus* occurrence in a particular area multiplied by 100. (**b**) The relationship between the percentage of validation occurrences on the vertical axis and the model prediction values on the horizontal axis. The red bars present the percentage of validation occurrence records for each prediction value (i.e., this shows that most validation records occurred in the upper 10th percentile of the predicted suitability values).

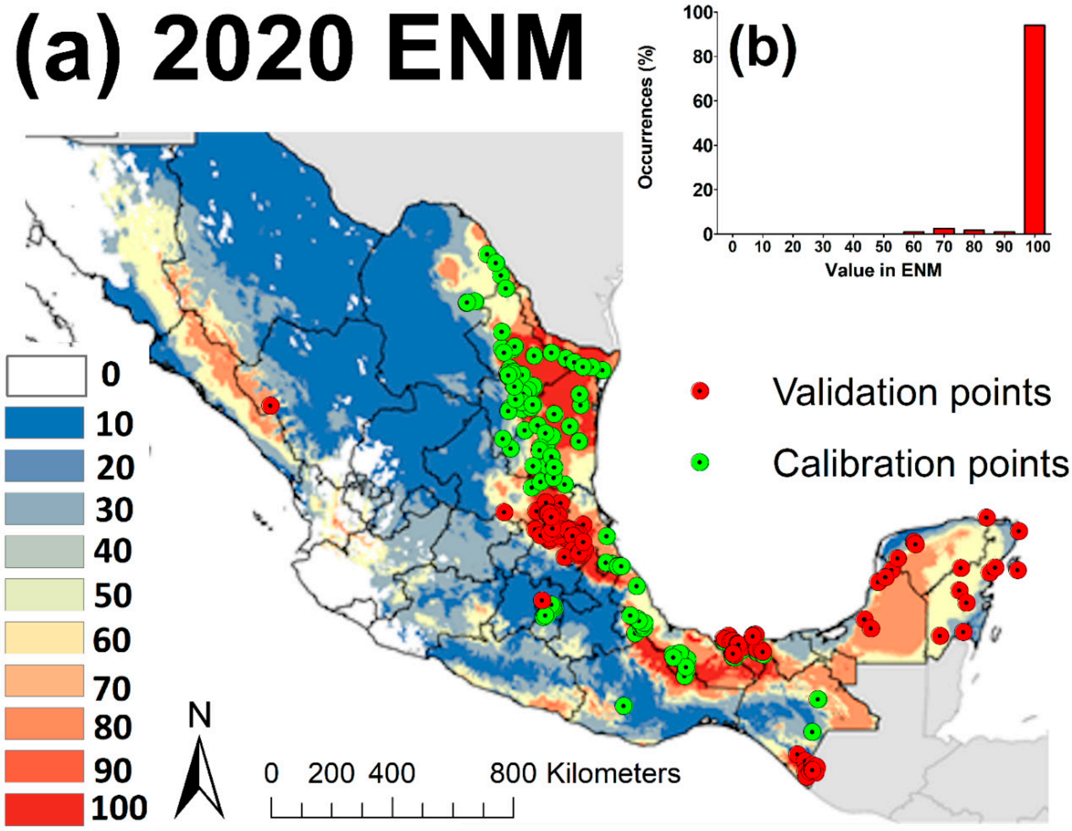

Supplement: Supplementary file 1 [file insects-12-00143-s001.pdf]
